# Development of Multi-Functional Graphene Polymer Composites Having Electromagnetic Interference Shielding and De-Icing Properties

**DOI:** 10.3390/polym11122101

**Published:** 2019-12-14

**Authors:** Ji-Hwan Ha, Soon-Kook Hong, Jae-Kwan Ryu, Joonwon Bae, Sung-Hoon Park

**Affiliations:** 1 Department of Mechanical Engineering, Soongsil University, 369 Sangdo-ro, Donjak-gu, Seoul 156-743, Korea; jhwan618@gmail.com; 2 Department of Mechanical and Naval Architectural Engineering, Naval Academy, Kyungsangnam-Do 440-749, Korea; hsk753@navy.mil.kr; 3 LIG Nex1, 207, Mabuk-ro, Giheung-gu, Yongin-si, Gyeonggi-do 13488, Korea; jaekwan.ryu@gmail.com; 4 Department of Applied Chemistry, Dongduk Women’s University, Seoul 02748, Korea

**Keywords:** graphene, polymer composite, EMI shielding, de-icing, heating unit

## Abstract

We developed a multi-functional graphene composite with electromagnetic interference (EMI) shielding and de-icing properties. Two-dimensional graphene fillers were homogeneously dispersed in a polymer by three-roll milling. The electrical properties and percolation threshold of the graphene composites were measured with various graphene contents. The variation in the EMI shielding properties of the graphene composites with respect to the filler content was measured. The shielding efficiency improved with increasing graphene filler content. Furthermore, we conducted electrical heating tests on the graphene composites. The composites could be heated rapidly to 200 °C by electrical Joule heating with low electric power because of the high electrical conductivity of the composite. Moreover, the composite film was suitable for application in a de-icing unit because of its rapid and homogenous heating performance.

## 1. Introduction

Because of their excellent mechanical strength and good electrical and thermal properties [1,2,3], carbon nanomaterials such as carbon black, carbon nanotubes, and graphene have been intensely investigated with respect to industrial applications in aircraft and vehicles [4,5,6,7,8,9,10]. Among these materials, graphene is often used as a filler in composites to improve both electromagnetic interference (EMI) shielding and the mechanical, electrical, and thermal properties. Graphene is a plate-like two-dimensional (2D) material with nanoscale thickness and microscale width. Because of its high aspect ratio, using it as a filler can create electrical and thermal networks [11]. Based on these properties, graphene-polymer composites have been prepared for various applications, such as conductive composite films or heating units [7,8]. In addition, 2D graphene networks in polymer composites can efficiently prevent un-wanted electromagnetic waves and are therefore promising as next-generation EMI shielding materials. 

However, carbon-based nanomaterials are easily aggregated because of the strong Van der Waals forces that bind them. To fabricate the ideal graphene composite, a uniform dispersion method is required to distribute the filler homogenously in the polymer matrix. The conventional method used is the ultra-sonication technique [12,13,14,15,16,17,18], where the filler is dispersed by ultrasonic waves. However, over time, the dispersed fillers seem to re-aggregate because of the attractive forces between them. To solve this problem, dispersants are used to stabilize dispersions; however, the dispersant causes deterioration of the electrical properties of the composite by damaging the filler surface. In addition, when the viscosity of the solution containing the fillers increases, the sonication method does not work. Therefore, other dispersion methods have been developed, such as the three-roll milling method, which uses mechanical forces [1,19]. This method does not require the use of a solvent and dispersant. Three-roll milling enables the homogenous dispersion of high contents of filler, regardless of viscosity. Therefore, well-dispersed composites can be prepared that can be suitable for many applications, such as heating units, micro-patterning heaters, flexible EMI shielding materials, and de-icing systems [20,21,22].

Conductive polymer composites offer some merits as EMI shielding materials. These include the low weight of the materials, corrosion resistance, and flexibility [23,24,25,26,27]. The flexibility of the composite is maintained by polymers such as epoxy and silicone elastomers. Carbon nanomaterials show excellent EMI shielding performances [28,29,30]; among carbon fillers, graphene has been extensively studied as a shielding material because of its unique 2D structure. Furthermore, these fillers have chemical stability and high conductivity, with a high aspect ratio. Table 1 shows the EMI shielding properties of graphene composites with various polymers. In previous works, graphene composites have been shown to have high EMI shielding effectiveness (SE) and flexibility [20,24,31,32,33]. For example, the EMI SE values of graphene-based composites with various polymers such as epoxy, polyetherimide (PEI), polymethylmethacrylate (PMMA), and polyurethane (PU) were 21 dB, 44 dB, 25 dB, and 16 dB, respectively [23,25,26,27]. Furthermore, a graphene polyvinylidene fluoride (PVDF) composite containing 7 wt% graphene showed EMI SE values of 20 dB in the 1–8 GHz frequency range and 25 dB in the 8–12 GHz range [24]. Moreover, a flexible graphene thin film had an SE of 20 dB at frequencies of 8–12 GHz [31]. Furthermore, graphene composites have superior EMI shielding properties compared with those of metal composites [26,33,34]. Carbon nanomaterial-based heating units have been developed by many researchers [35,36,37,38]. These heaters can be heated efficiently by Joule heating. Electric Joule heating is facilitated by phonons colliding with electrons from an applied electric field [39,40,41], as a result of which an electro-thermal response is generated. Conductive composites containing fillers such as carbon nanotube (CNT) and graphene are particularly efficient for this process because of their high electrical conductivities. Among these composites, graphene composite-based heating units have been extensively studied [41,42]. With respect to the selection of the polymer, a conductive composite can be made suitable for flexible heating units by exploiting the flexibility of the polymer matrix. At the same time, the rate of heating of the composite is high because of the outstanding electrical and thermal conductivity of graphene [43]. Graphene composites are used in various applications, such as curved heaters, patterned heaters, and de-icing units, because of their flexibility and rapid heating capability [22,41].

In this paper, we describe the fabrication of graphene polydimethylsiloxane (G-PDMS) composite films using a three-roll mill process. We measured the electrical properties of the composites while varying the filler content. Furthermore, EMI shielding and electrical heating performances were evaluated. We confirmed the possibility of application of the composites for EMI shielding and de-icing units, based on these properties. The composite shows outstanding EMI shielding and de-icing performance as a result of the homogeneous distribution of the filler. Thus, we show that graphene composite films can be applied in electric vehicles and in the aviation industry, where both EMI shielding and de-icing properties are simultaneously required.

## 2. Materials and Methods 

### 2.1. Material Preparation

We fabricated a G-PDMS composite film for EMI shielding and de-icing applications. Fillers were purchased from Angstron Materials Inc. (graphene powder N006-P). Graphene powder comprised flakes that were 10–20 nm in thickness and measured 5.0 μm in the x-y directions. PDMS (Sylgard 184) was used as the base polymer of the composite film. First, for preparing the composite paste, Sylgard 184 (10:1) and graphene were premixed using a paste mixer (500 rpm for 30 s, 1500 rpm for 60 s). After the premixing step, we used a three-roll milling machine (Intech, TX-3102) to homogeneously disperse the fillers by applying mechanical shear forces. The G-PDMS paste was dispersed with the help of a three-roll milling machine at 100 rpm for 6 min. 

### 2.2. Fabrication of a G-PDMS Film

Graphene-dispersed G-PDMS paste was used to form a film of 500 μm thickness using hot press-machine; this technique yielded a film with uniform thickness. The paste was cured at 150 °C for 40 min to achieve stable curing. The pressure of the machine was set at 15 MPa for film fabrication.

### 2.3. Measurement of EMI Shielding

We measured the EMI SE of the G-PDMS film using a network analyzer (E5061B, Agilent Technologies) with an S-parameter. A circular sample with 1 mm thickness and a diameter of 10 cm was fabricated; a reference sample with the same dimensions was also prepared. The SE value of the composite was measured (based on the ASTM D4935-99 method) in the 1.0–3.0 GHz frequency range.

### 2.4. De-Icing Experiment

The electrical heating properties of G-PDMS were characterized for de-icing unit application. Silver and copper electrodes were formed on the film. The silver electrode was made using silver paste; after curing the silver paste at 175 °C for 1 h, copper was attached to the silver electrode of the composite film. To heat the film, we used a power supply (EX80-5, ODA technology, Incheon, Korea) to input electrical power to the G-PDMS. An IR camera was used to measure the surface temperature of the film. In de-icing tests, a cooling spray was used to control the low temperature of −50 °C.

## 3. Results and Discussion

**Figure 1:**
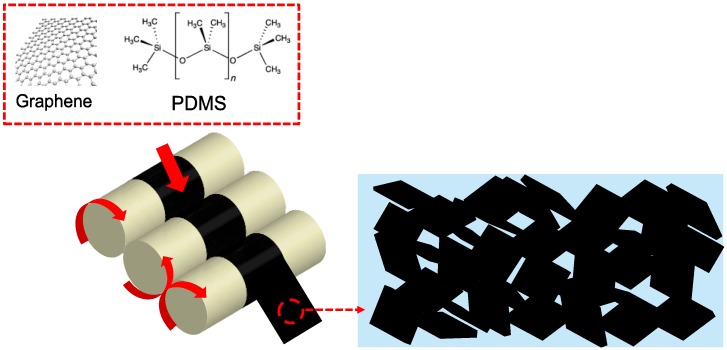
Schematic showing the fabrication of a graphene polydimethylsiloxane (G-PDMS) composite using the three-roll milling method. The 2D carbon nano fillers are dispersed by the mechanical shear forces of the rolls.

We fabricated G-PDMS composites for application in EMI shielding and in a de-icing unit. The composite film was produced using a three-roll mill. Nano-sized fillers usually aggregate because of the Van der Waals forces; hence, special methods are required obtain a homogeneous dispersion of graphene so that its excellent material properties can be exploited. Therefore, we used a solvent-less dispersion method that makes use of mechanical shear forces. The fabrication process of the graphene dispersion is shown in Figure 1. This process can be used to obtain composites with varying graphene contents, from low (<0.5 wt%) to high (up to 20 wt%). In addition, the micro-scale gaps between the rolls enable a homogeneous dispersion of G-PDMS because of the high shear force. Using the three-roll milling technique, a uniformly dispersed paste containing numerous fillers can be obtained. Furthermore, the paste does not re-aggregate after the process because of the high viscosity of PDMS. We fabricated G-PDMS with varying filler contents; graphene composites were produced with 1.1 vol%, 2.3 vol%, and 3.6 vol% of graphene, and their performances in EMI SE and de-icing were evaluated. Table 2 shows the PDMS-based composite that we fabricated. We refer to them as the low graphene-containing composite (LG-PDMS; 1.1 vol%), middle graphene-containing composite (MG-PDMS; 2.3 vol%), and high graphene-containing composite (HG-PDMS; 3.6 vol%), respectively.

**Figure 2:**
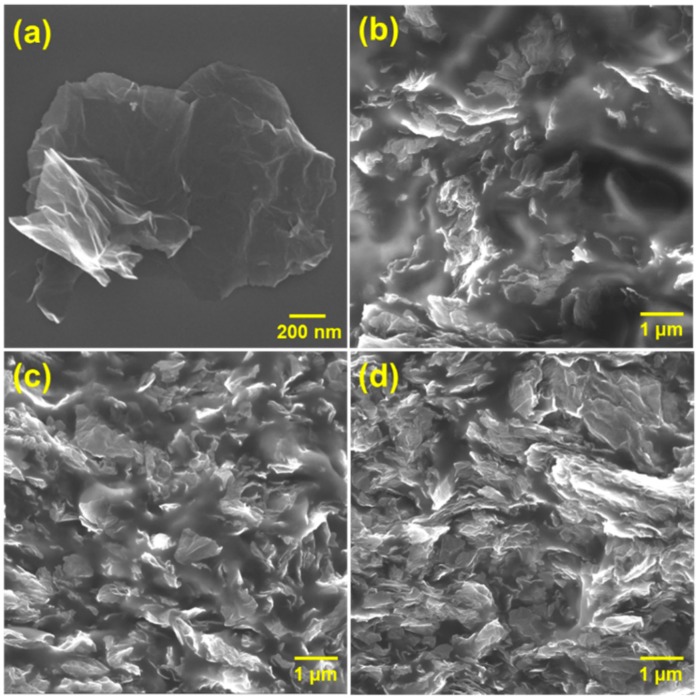
SEM image of (**a**) graphene. Cross-section SEM view of (**b**) low graphene (LG)-PDMS, (**c**) middle graphene (MG)-PDMS, and (**d**) high graphene (HG)-PDMS. Graphene is dispersed homogeneously in the polymer.

We observed the inside morphology of the graphene composite using scanning electron microscopy (SEM). Figure 2 shows the distribution of graphene and the morphology of the composite. Frist, we confirmed the size of the graphene itself to be in the range of 3–4 μm, as shown Figure 2a. Graphene was found to have a high aspect ratio with nanoscale thickness and microscale width. In the case of G-PDMS, fracture SEM images (Figure 2b,c) show that the filler distribution is in a random direction arrangement in the composite matrix.

**Figure 3:**
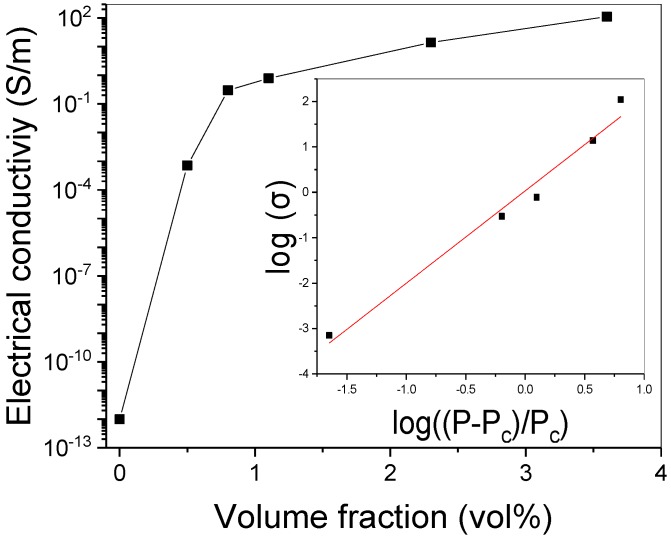
Electrical conductivity of the G-PDMS composite as a function of graphene vol% and electrical percolation threshold of a G-PDMS composite. Inset: log-log plot of the conductivity of the composite according to the relation ((P − P_c_)/P_c_); (P_c_ = 0.48 vol%, β = 3.9)**.**

In addition, the morphology of G-PDMS indicates a homogeneously dispersed state without aggregation of fillers. For HG-PDMS, a sufficiently dense distribution of the fillers in the interior of the composite can be seen. The electrical properties of G-PDMS composites were measured for various filler contents. Pure PDMS is a non-conducting polymer; the addition of a small amount of graphene can improve the electrical properties of the composite because of the superior electrical conductivity and high aspect ratio of the filler. In Figure 3, following the initial increase in filler content to 1.2 vol%, a very sharp increase in conductivity is observed. We also fabricated G-PDMS with lower filler contents (0.5 vol% and 0.8 vol%) than LG-PDMS to observe the electrical percolation threshold phenomenon of the composite. After 1.2 vol%, the conductivity increases only gradually, showing saturation. The electrical conductivity of the HG-PDMS composite increased to 110 S/m. We observed electrical percolation behavior (transition from insulator state to conducting state) following a power law of the form [44,45]:

σ ∝ (P − P_c_)^β^ (1)

This equation suggests that the conductivity increases when the filler volume fraction increases. Furthermore, the results of the equation are known as the electrical percolation threshold tendency, where σ is the composite conductivity, P_c_ is the volume fraction (P) of the electrical percolation threshold, and β is a critical exponent. From the best fit of log-log plots of the power low (inset Figure 3), P_c_ (0.48 vol%) and β (3.9) are obtained. The P_c_ and β values are quite similar to those of a previously reported graphene composite (P_c_ and β are 0.5 vol% and 1.31) [46].

**Figure 4:**
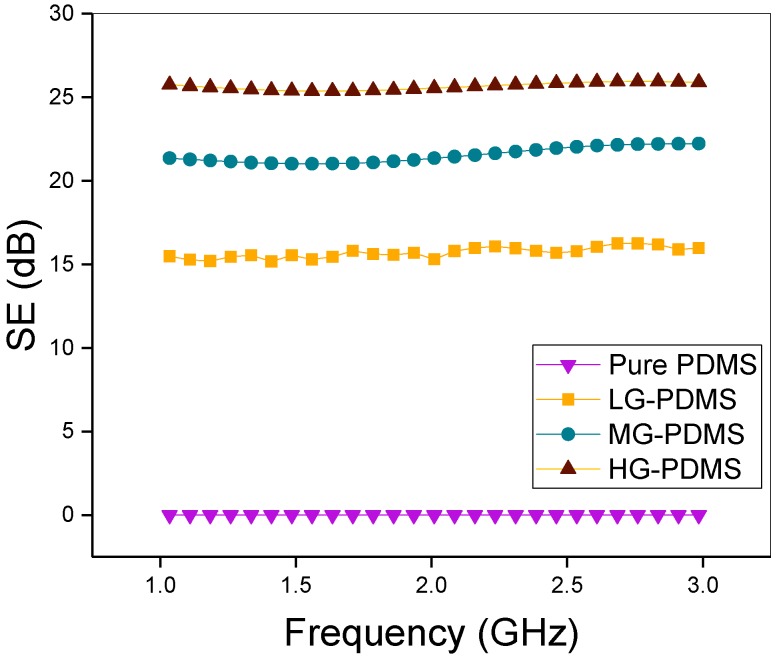
EMI shielding effectiveness (SE) of G-PDMS composites with various filler contents (1.1 vol%, 2.3 vol%, 3 vol%, and pure PDMS); the frequency range is 1.0–3.0 GHz.

In Figure 4, the EMI SE data of G-PDMS with respect to filler content are shown. The EMI SE was calculated as follows [20,23,30]:

SE = 10 logP_i_/P_t_ (2)

EMI SE is calculated with P_i_ and P_t_ data measured by the network analyzer (Equation (2)), where P_i_ and P_t_ are the magnitudes of the incident and transmitted power densities, respectively. The EMI SE of the graphene composite is measured over the frequency range of 1.0–3.0 GHz. Generally, pure PDMS does not show EMI shielding properties. We measured the EMI SE values of graphene composites. When graphene filler is added to the polymer, the EMI SE increases. For the LG-PDMS composite, an EMI SE value of 15 dB is measured. Furthermore, the EMI SE value of the MG-PDMS graphene film improved to 21 dB. Finally, for the composite with the highest graphene content (3.6 vol%, 8 wt%), the EMI SE value increased to ~25 dB in the frequency range 1.0–3.0 GHz. Because of the sufficiently high values of the EMI SE at low graphene contents, G-PDMS can be applied for EMI shielding in electric vehicles and aircraft. 

**Figure 5:**
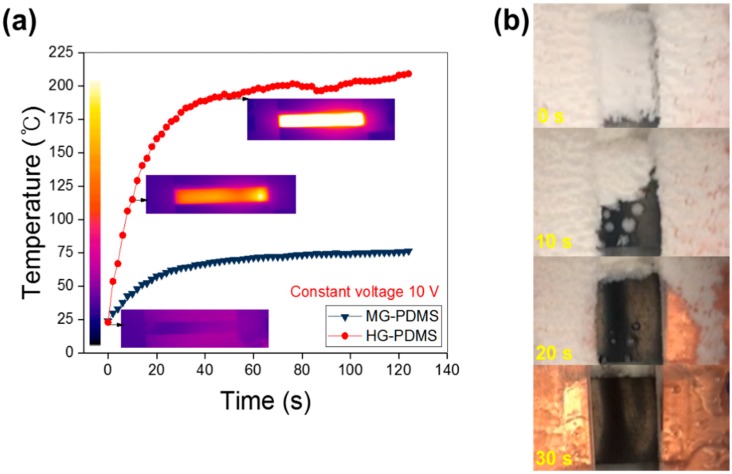
Electrical heating performance of G-PDMS according to filler contents at constant voltage (10 V). (**a**) MG-PDMS film can induce rapid heating performance from room temperature by electrical power. (**b**) De-icing properties of the HG-PDMS heating unit are superior because of its rapid heating properties.

For application of the composite in a de-icing unit, we performed de-icing experiments by electrical heating. Graphene composites are used as heating units because of their rapid heating performance resulting from the presence of carbon-based fillers that have superior electrical and thermal properties. When the graphene fillers are well-dispersed, the surface temperature is increased homogeneously upon application of electric power. These properties of the graphene composite enable rapid and uniform heating units to be fabricated. Figure 5a shows the electrical heating properties of MG-PDMS and HG-PDMS. The surface temperature of the MG-PDMS heater increased from room temperature (26 °C) to 70 °C in 50 s, when 10 V of power was applied. The LG-PDMS composite was not heated by 10 V of power because of its low conductivity. However, the HG-PDMS heating unit was heated higher than the MG-PDMS because of the high contents of graphene. The maximum surface temperature of this composite is 200 °C at constant voltage. As described by the IR images, the composite in the form of a film is heated uniformly at 200 °C because of the homogeneously dispersed state of the film. Moreover, this heating unit can be heated rapidly from room temperature to 200 °C in 50 s, which makes it suitable for a quick de-icing system.

Figure 5b shows the ice removal properties of the graphene heating unit. The ice on the graphene composite surface was formed at a temperature of −50 °C. Following electric power input to G-PDMS, the surface temperature increased sharply, and the ice on the film surface was removed in 30 s. In view of this rapid de-icing performance, the unit based on the graphene composite can be used in de-icing applications.

## 4. Conclusions

Graphene-based composites have been investigated for various applications, such as EMI shielding and de-icing. We fabricated G-PDMS composites by three-roll milling. The fillers were dispersed homogeneously inside the composite, by which the electrical conductivity of the graphene composite improved to 10^2^ S/m and the EMI SE was improved to levels required for application in EMI shielding systems. The SE of the flexible composite was 25 dB in the 1.0–3.0 GHz frequency range; this EMI SE value indicates possible application of the composites in electromagnetic wave shielding. In addition, the G-PDMS unit can be heated rapidly (from room temperature to 200 °C in 50 s) to enable fast de-icing. Thus, in view of its superior EMI SE and rapid heating performance, the composite material is suitable for EMI and icing prevention units and can be applied in vehicles and the aviation industry.

## Figures and Tables

**Table 1:** Previous studies of graphene polymer composites for electromagnetic interference (EMI) shielding properties.

Composite	Graphene Content	EMI SE	Ref.
Graphene/Epoxy	15 wt%	21 dB	[23]
Graphene/PVDF	7 wt%	20 dB	[24]
Graphene/PEI	10 wt%	44 dB	[25]
Graphene/PMMA	5 wt%	25 dB	[26]
Graphene/PU	0.3 wt%	16 dB	[27]

**Table 2:** G-PDMS composite samples according to graphene contents.

Sample	Graphene Volume Fraction	Graphene Weight Fraction	3-Roll Milling Time
Pure PDMS	0 vol%	0 wt%	0 min
LG-PDMS	1.1 vol%	2.5 wt%	6 min
MG-PDMS	2.3 vol%	5 wt%	6 min
HG-PDMS	3.6 vol%	8 wt%	6 min
